# Genome-Wide Identification, Evolutionary Analysis, and Expression Profiling of the β-D-Xylosidase Gene Family in Cotton (*Gossypium hirsutum*) Under PEG-Simulated Osmotic and Salt Stress

**DOI:** 10.3390/biology15161419

**Published:** 2026-08-18

**Authors:** Zhenzhen Wei, Anxing Zhu, Yang Liu, Fangjie Xiong, Zhi Wang, Yihan Xue, Fei Wei

**Affiliations:** 1 State Key Laboratory of Cotton Bio-Breeding and Integrated Utilization, Institute of Cotton Research, Chinese Academy of Agricultural Sciences, Anyang 455099, China; wzz19920315@163.com (Z.W.); liuyang7767@126.com (Y.L.); xiongfj2009@126.com (F.X.); wangzhi01@caas.cn (Z.W.); 2 Key Laboratory of Plant-Soil Interactions of MOE, College of Resources and Environmental Sciences, National Academy of Agriculture Green Development, China Agricultural University, Beijing 100193, China; zax13051521265@163.com; 3 College of Life Sciences, Luoyang Normal University, Luoyang 471934, China; 19712893626@163.com

**Keywords:** *Gossypium hirsutum*, β-D-xylosidase, BXL gene family, abiotic stress, expression profiling

## Abstract

β-D-Xylosidases (BXLs) are members of glycoside hydrolase family 3. They play essential roles in cell wall remodeling, plant development, and abiotic stress responses. BXL gene families have been systematically characterized in several crop species. However, a comprehensive analysis of *BXL* genes in cotton (*Gossypium hirsutum*), a globally important fiber and oilseed crop, is still lacking. In this study, we performed a genome-wide identification of *BXL* genes in allotetraploid cotton. A total of 25 *GhBXL* genes were identified and classified into six phylogenetic clades. Gene structure and conserved domain analyses showed that all GhBXL proteins possess the characteristic tripartite GH3 domain architecture. Chromosomal distribution and synteny analyses indicated that the expansion of the cotton BXL family may be associated with whole-genome duplication and allopolyploidization. Promoter *cis*-element analysis detected stress-responsive regulatory motifs in the *GhBXL* promoters, including STRE, W-box, DRE core, and as-1 elements. Under PEG-simulated drought and salt stress, expression profiling, independently confirmed by qRT-PCR, showed distinct temporal response patterns among GhBXL members. Weighted gene co-expression network analysis (WGCNA) further identified *GhBXL-8*, *GhBXL-9*, and *GhBXL-20* as hub genes in stress-responsive modules. Their co-expressed partners were enriched in transcription factors, kinases, and stress-related proteins. These findings provide a systematic foundation for understanding the evolutionary dynamics and functional roles of BXL genes in cotton. They also highlight candidate genes for future functional investigation.

## Simple Summary

Cotton is one of the world’s most important fiber and oil crops, but drought and salty soils strongly reduce its growth and yield. Enzymes of the BXL family help remodel plant cell walls and are thought to contribute to a plant’s ability to cope with environmental stress. In this study, we searched the cotton genome and identified 25 BXL genes, and we examined their evolutionary relationships, gene structures, and the regulatory regions that control their activity. We then tested how these genes respond when cotton seedlings are exposed to drought-like and salt-like conditions. Several BXL genes were strongly activated under both treatments, and three in particular were identified as central “hub” genes within networks of stress-responsive genes. These results indicate that specific BXL genes may help cotton survive dry or salty conditions, and they provide candidate targets for future efforts to improve cotton’s tolerance to drought and salinity. Such improvements could help stabilize cotton production under increasingly challenging environmental conditions.

## 1. Introduction

The functional landscape of β-D-xylosidases (BXLs) spans critical aspects of plant life, ranging from developmental regulation to stress adaptation. In terms of growth and development, BXLs act as key determinants of organ morphology; for instance, the stem-specific AtBXL1 in Arabidopsis governs stem elongation and seed yield [1]. Their role extends to fruit physiology, where they modulate senescence in pear and confer chilling tolerance in peach (PpXy1) [2,3]. Regarding hormonal and environmental signaling, BXL expression is tightly orchestrated by external cues: sugar starvation and darkness upregulate AtBXL1 [1,4]; hormone treatments suppress FaXyl1 in strawberry [5]; and nitrogen availability induces specific PtBXL members in poplar [6]. Furthermore, AtBXL4 has been established as a pivotal component of the systemic acquired resistance (SAR) pathway in the apoplast [7].

Most notably, emerging evidence highlights the central role of BXLs in abiotic stress resilience across diverse crops. While rice OsBXL1 and OsBXL3 are known to respond to salt-alkali stress [8], recent genome-wide analyses have significantly broadened this understanding. In soybean (*Glycine max*), a conserved systemic salt-tolerance mechanism was identified, where 20 out of 29 *GmBXL* genes were coordinately upregulated in both roots and leaves under salt stress [9]. Conversely, functional characterization in potato (*Solanum tuberosum*) revealed a negative regulatory role, with StBXL4 overexpression exacerbating seedling sensitivity to alkaline stress, thereby identifying it as a susceptibility factor [10]. Collectively, these findings illustrate that BXLs are not merely hydrolytic enzymes but versatile regulators integrating developmental cues and stress signals. Although systematic identification has now covered major legumes and tubers, dissecting the precise molecular mechanisms by which specific BXL isoforms—such as the contrasting regulators in soybean and potato—mediate stress tolerance remains a critical frontier for future research.

As a top global cash crop, cotton provides the textile industry with vital natural fibers and supports the incomes of more than 100 million families worldwide [11]. However, its value goes far beyond fiber; cotton is a key part of the world’s food supply. About 65% of the total cotton harvest is used for food, either directly as cottonseed oil or indirectly when farm animals eat cottonseed meal and ginning leftovers to produce meat and dairy [12,13,14].

However, environmental stresses caused by climate change, especially drought and high salt levels, are becoming growing threats to cotton production. A lack of water during the reproductive stage is particularly harmful. It causes flowers to drop early, stops bolls from growing properly, and greatly reduces both the number and size of bolls, leading to major yield losses [15,16,17,18,19]. Although young plants can handle some stress, the flowering and boll-forming stages are very sensitive to low water availability [20]. Similarly, salt stress creates two main problems: it makes it hard for plants to take in water and introduces toxic ions. This blocks water uptake, closes leaf pores, lowers photosynthesis, and upsets the balance of minerals, especially causing a lack of potassium. It also triggers cell damage that requires strong defense systems to fix [21,22,23]. Since these environmental challenges seriously hurt crop growth, fiber quality, and stable yields, finding and using genes that help cotton resist stress is essential for developing stronger varieties and ensuring sustainable farming.

Although BXL genes have been linked to abiotic stress in several species, the BXL family of cotton remains poorly characterized. In this study, we performed genome-wide identification, phylogenetic analysis, gene structure and conserved domain analysis, chromosomal localization and synteny analysis, promoter cis-element analysis, as well as expression profiling under drought and salt stress of the BXL gene family in *G. hirsutum*. Through transcriptomic expression profiling combined with qRT-PCR confirmation, we found that *GhBXL-8*, *GhBXL-9*, and *GhBXL-20* may possess potential roles in salt and drought tolerance. These findings provide a systematic foundation for understanding the evolutionary dynamics and functional roles of *BXL* genes in cotton, and also suggest candidate genes that merit further functional investigation.

## 2. Materials and Methods

### 2.1. Retrieval and Identification of Putative BXL Proteins

To identify BXL family members, we combined hidden Markov model (HMM) searches, InterProScan validation, and BLASTP anchoring. Three Pfam profiles corresponding to the two catalytic domains of glycoside hydrolase family 3 (PF00933, GH3 N-terminal domain; PF01915, GH3 C-terminal domain) and the fibronectin type III-like domain that distinguishes beta-xylosidases from other GH3 members (PF14310) were retrieved from Pfam and searched against the proteomes of 39 plant species using hmmsearch (HMMER v3.1b2) with an E-value cutoff of 0.01; only proteins carrying all three domains were retained [24,25]. Domain architecture was then verified with InterProScan, and candidates were retained as BXL members when they contained the Pfam signatures PF00933, PF01915, and PF14310 [26]. To independently anchor the candidates to the beta-xylosidase lineage, the seven characterized *A. thaliana* beta-D-xylosidases (AtBXL1-7) were used as BLASTP queries against each species’ proteome (E-value ≤ 1 × 10^−20^). All 430 retained proteins were recovered as AtBXL homologs (100% at ≥30% identity; 82.8% at ≥60% identity). For each species, only the longest protein isoform per gene model was retained. Final membership was confirmed phylogenetically by placing the candidates together with AtBXL1-7 and non-BXL GH3 reference sequences on a maximum-likelihood tree (Figure S1). Basic physicochemical properties were calculated using the ProtParam tool on the ExPASy server [27]. The 25 cotton members were then designated GhBXL-1 to GhBXL-25 in order of their physical positions along the A- and D-subgenome chromosomes.

### 2.2. Sequence Alignment and Phylogenetic Analysis

The amino acid sequences of 102 BXL proteins from nine representative species (spanning eudicots and monocots) were aligned with MAFFT v7.467 using the –auto strategy, which selected the FFT-NS-i algorithm (BLOSUM62, gap-opening penalty = 1.53, gap-extension penalty = 0.123) [28]. Poorly aligned and phylogenetically uninformative regions were removed with BMGE v1.12 (entropy cut-off = 0.5, gap-rate cut-off = 0.5, sliding window = 3, minimum block size = 5), and the curated alignment contained 546 amino-acid positions after BMGE trimming (from 2806 initially) [29]. A maximum-likelihood phylogenetic tree was then inferred from the curated alignment using FastTree v2.1.11 under the LG substitution model with Gamma-distributed rate heterogeneity (-lg -gamma; estimated alpha = 0.795), and branch support was assessed using the SH-like approximate likelihood-ratio test (SH-aLRT) rather than bootstrap resampling [30]. Pairwise protein-sequence similarity was estimated under the LG+Γ substitution model. The tree was visualized and annotated using the TVBot online platform [31]. The tree was left unrooted; the six major clades (A–F) were delimited by SH-aLRT-supported (≥80%) monophyletic groups at the deepest internal branches.

### 2.3. Gene and Protein Structure Analysis

Exon information was extracted directly from the genome annotation files. Conserved protein domains were identified and marked using InterProScan [26]. For *cis*-acting element analysis, 2000 bp DNA sequences upstream of the transcription start site of each *GhBXL* gene were retrieved. Potential regulatory elements in these regions were detected using the PlantCARE database [32]. To assess statistical enrichment, the frequency of each motif in the *GhBXL* promoters was compared against the background of all cotton gene promoters using Fisher’s exact test with Benjamini–Hochberg FDR correction. Per-gene motif counts are listed in Table S3. Finally, gene structures, including exon distribution, conserved domains, and *cis*-acting elements, were combined and visualized using TBtools (v.2490) [33].

### 2.4. The Analysis of Chromosome Location and Gene Collinearity

MCScanX was used to analyze chromosome positions and duplication events based on genome annotation files (protein sequences and GFF files). Collinear gene pairs within the BXL family were identified, and BXL-related duplication events were detected by this program [34]. Syntenic relationships were shown in circular plots generated by Circos [35].

### 2.5. Transcriptome Analysis of BXL Stress Response

The RNA sequencing (RNA-Seq) dataset used in this study was retrieved from the Sequence Read Archive (SRA) database under accession number PRJNA490626. This dataset corresponds to transcriptomic profiling of cotton plants subjected to salt and PEG stress treatments at five key time points (1, 3, 6, 12, and 24 h post-treatment). Prior to subsequent genomic alignment, raw sequencing reads were quality-trimmed, and low-quality sequences were eliminated using the Trimmomatic tool to obtain high-quality clean reads [36]. The resultant clean reads were then aligned against the cotton reference genome via HISAT2 software [37]. Gene transcript assembly was performed with StringTie, and the fragments per kilobase of transcript per million mapped reads (FPKM) values of all genes were calculated using R programming language to quantify gene expression levels [38].

The RNA-Seq dataset used in this study was retrieved from the NCBI Sequence Read Archive (SRA) under BioProject PRJNA490626. The dataset comprises transcriptome profiles of four-week-old *Gossypium hirsutum* cv. TM-1 seedlings subjected to abiotic stress. For salt stress, seedlings were exposed to 400 mM NaCl; for drought stress, seedlings were treated with 20% (*w*/*v*) PEG-6000. Leaf tissues were collected at 1, 3, 6, 12, and 24 h post-treatment, with three biological replicates per time point. Seedlings were grown under controlled greenhouse conditions (200 µmol m^−2^ s^−1^ light intensity, 16/8 h photoperiod, 28 ± 2 °C day/night). Raw reads were quality-filtered with Trimmomatic (default settings) to obtain clean reads, which were aligned to the cotton reference genome (ZJU2.1) using HISAT2. Transcript assembly and FPKM quantification were performed with StringTie, and gene expression levels were calculated in R 4.5.2 [36,37,38].

Differentially expressed genes (DEGs) were identified with edgeR using raw read count matrices, with |logFC| > 1 and *p* < 0.05 as thresholds [39]. Weighted gene co-expression networks were constructed with the WGCNA package. A soft-threshold power β = 18 was selected by pickSoftThreshold to approximate a scale-free topology, under an unsigned co-expression network, with a minimum module size = 30 and module-merge cutHeight = 0.25; modules were divided by dynamic tree cutting on the topological-overlap dissimilarity matrix (dissTOM = 1 − TOM) [40]. Hub genes were defined as the most highly connected members of each module on the basis of intramodular connectivity. Finally, the core co-expression network visualization was conducted using Cytoscape software 3.10.2 [41].

### 2.6. RNA Extraction and RT-qPCR Analysis

TM-1 cotton (*G. hirsutum*) seeds were germinated in a growth chamber under a 16/8 h light/dark cycle at 27 °C/22 °C. At the three-leaf stage, seedlings were treated with 200 mM NaCl or 10% (*w*/*v*) PEG-6000. Leaf tissues were harvested at 0 (control), 1, 3, 6, 12, and 24 h after treatment, with three biological replicates per time point. All samples were immediately frozen in liquid nitrogen and stored at −80 °C. Total RNA was isolated using the FastPure Plant Total RNA Isolation Kit (Vazyme). Genomic DNA was removed, and first-strand cDNA was synthesized using HiScript III RT SuperMix (Vazyme). RT-qPCR reactions were performed with SYBR qPCR Master Mix (Vazyme, Cat. No. Q711-02). The cotton ubiquitin gene (*GhUBQ*) was used as an internal reference for normalization. Relative expression levels were determined using the 2^−ΔΔCt^ method [42]. Primer sequences are provided in Supplementary Table S2. The qRT-PCR validation was performed at reduced treatment intensities (200 mM NaCl; 10% (*w*/*v*) PEG-6000) relative to the RNA-Seq experiment (400 mM NaCl; 20% (*w*/*v*) PEG-6000) and therefore serves as an independent confirmation of stress responsiveness rather than a direct replicate of the transcriptomic time course. For statistical analysis, the non-parametric Kruskal–Wallis test followed by Dunn’s multiple comparisons test was applied (α = 0.05).

## 3. Results

### 3.1. Evolution and Expansion of the BXL Gene Family

**Figure 1:**
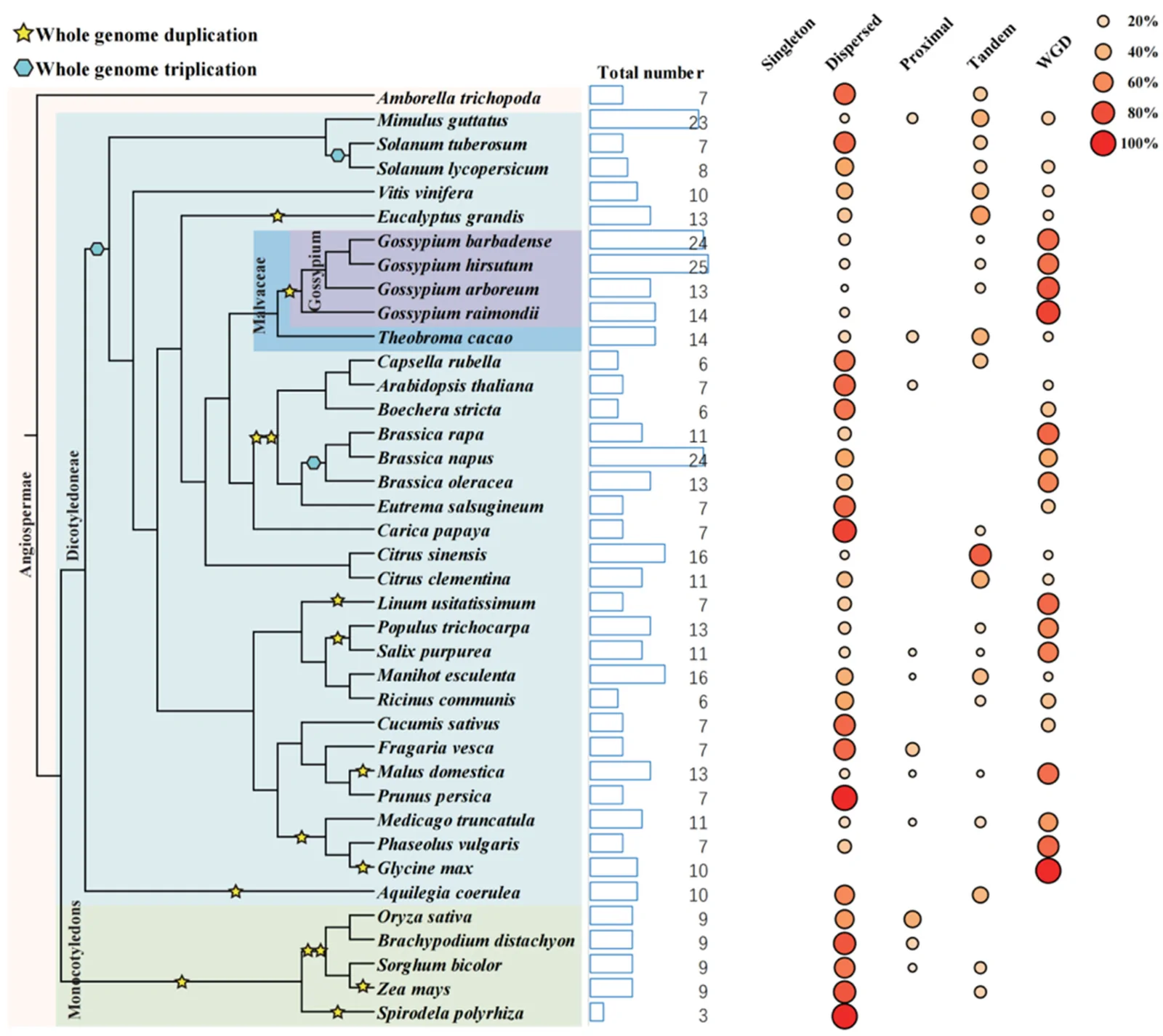
Evolutionary expansion patterns of the BXL gene family across 39 plant species. The phylogenetic tree on the left illustrates the evolutionary relationships among studied species, with star symbols indicating documented whole-genome duplication (WGD) or whole-genome triplication (WGT) events. The species tree on the left was adopted from the TimeTree of Life database (https://timetree.org). The central column displays the total number of BXL genes per species. The right panel uses proportional bubble charts to show the relative contribution of five duplication modes including singleton, dispersed, proximal, tandem, and WGD to BXL gene family expansion in each lineage, with color intensity reflecting the percentage contribution from 20% to 100%.

The BXL gene family is present in all 39 plant species examined, ranging from ancient lineages to modern monocots and dicots, indicating its widespread conservation across the plant kingdom (Figure 1). However, the gene copy number varies significantly among taxa. Monocot plants (e.g., rice and maize) typically possess a limited number of *BXL* genes (3–9). In contrast, dicot plants exhibit greater variability, with gene counts ranging from 6 to 25. Furthermore, the expansion mechanisms of the BXL family differ markedly across species. The monocot species analyzed generally lack recent whole-genome duplication (WGD) events influencing this family. Conversely, WGD events are prevalent in dicots. Among different species, the dominant mode of expansion varies: some may be associated with dispersed duplication, others by tandem duplication, whereas specific lineages, such as cotton, are dominated by WGD events (Figure 1).

Notably, our analysis reveals that the expanded *BXL* repertoire in upland cotton is predominantly attributed to WGD, and the allotetraploid cotton species, *G. hirsutum* and *G. barbadense*, possess a number of *BXL* genes that is nearly equivalent to the sum of those found in their diploid ancestors (*G. arboreum* and *G. raimondii*). A strikingly similar pattern is observed in *Brassica napus*, where the BXL gene count also approximates the combined total of its two ancestral genomes (*B. rapa* and *B. oleracea*), confirming that this expansion might be driven by the same mechanism of allotetraploidy (Figure 1). Collectively, these findings suggest that allotetraploidy also serves as a primary evolutionary driver for the substantial expansion of the BXL gene family in allotetraploid crops.

### 3.2. Phylogenetic Analysis of BXL Proteins

**Figure 2:**
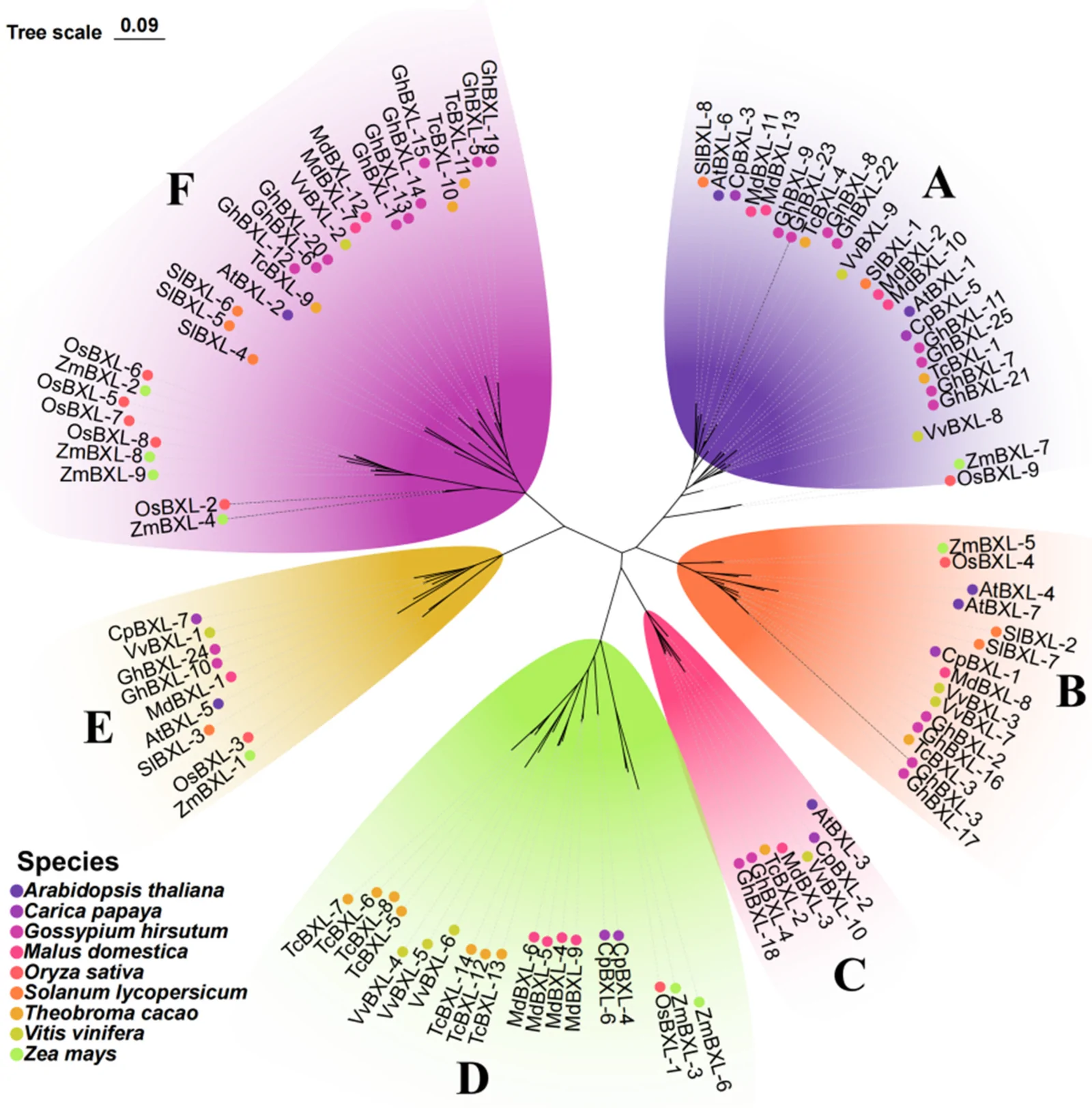
Phylogenetic relationships and subfamily classification of the BXL gene family. An unrooted tree based on full-length BXL proteins from nine representative plant species (scale bar = 0.09) groups genes into six color-coded clades (**A**–**F**). Gene names are labeled at the tips, with colored circles indicating BXL proteins from different species.

To elucidate the evolutionary relationships within the BXL gene family, a phylogenetic tree was constructed using full-length protein sequences from nine representative plant species (Figure 2). The analysis resolved the *BXL* genes into six distinct clades, each highlighted with a unique background color to denote potentially evolutionarily conserved subfamilies. The distribution of genes across these clades reveals a complex pattern of diversification: while some subfamilies contain members from a broad range of species including monocots and eudicots, this might suggest ancient origins, potentially predating the monocot–eudicot split, whereas others appear to exhibit lineage-specific expansions. No BXL members from *Gossypium hirsutum* were identified in Clade D, which could suggest either a lineage-specific loss of this subfamily in cotton or that it was never present in this lineage. Given the unrooted gene tree, these alternatives remain tentative and will require broader Malvaceae sampling to be resolved. Notably, Clade F is dominated by *G. hirsutum* (9/28, 32.1%) and also includes a substantial number of monocot genes (9/28, 32.1% combined from rice and maize), suggesting lineage-specific expansions in cotton and grasses. In addition, Clade C lacks monocot representatives, suggesting either an eudicot-specific retention or a monocot-specific loss of this subfamily. Conversely, certain clades show sparse representation from specific species, possibly reflecting gene loss or differential retention during the evolution (Figure 2).

### 3.3. Structural Characteristics and Conservation Analysis of BXL Genes

**Figure 3:**
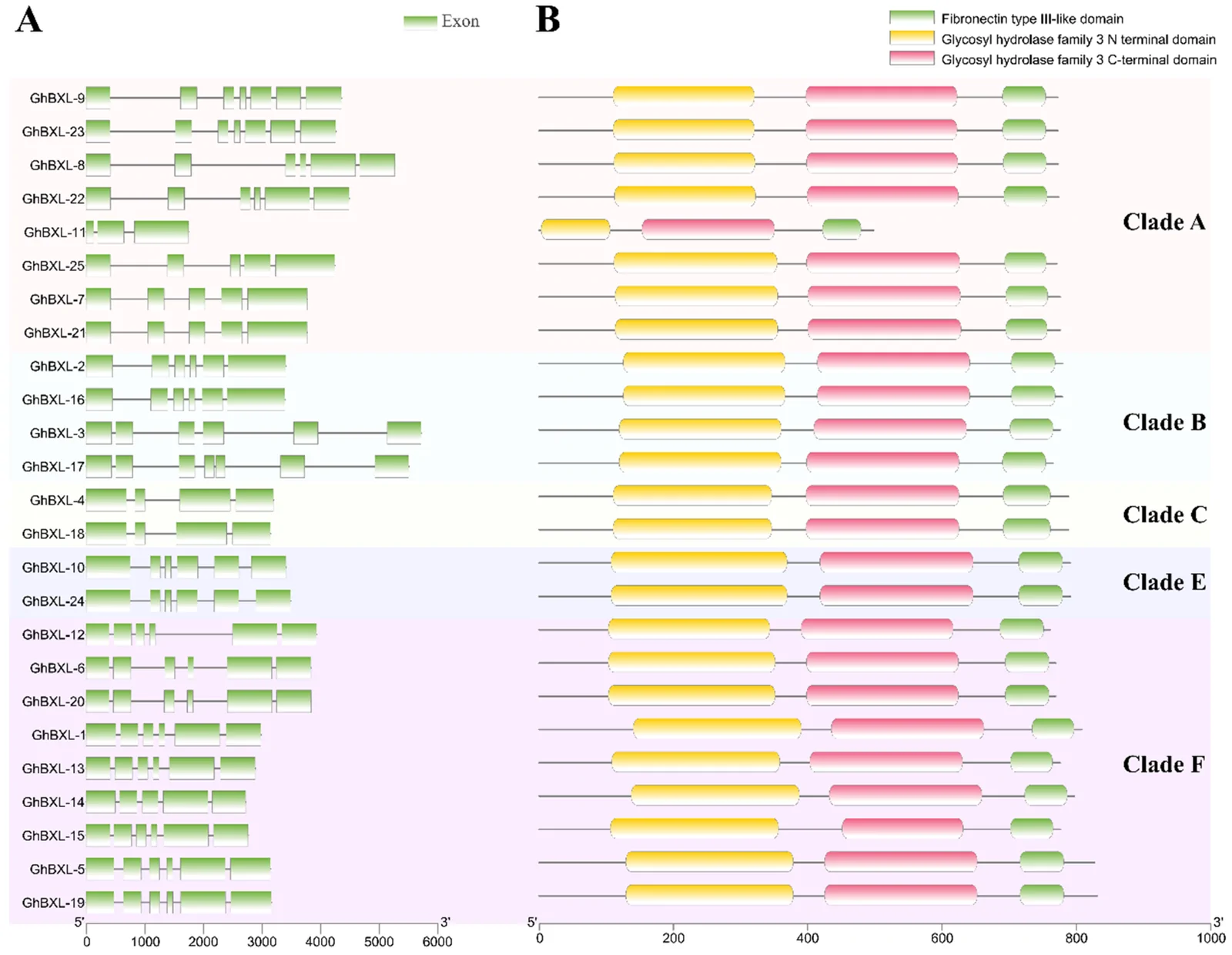
Structural characterization of the GhBXL gene family in cotton. (**A**) Exon–intron architecture of 25 *GhBXL* genes. Green boxes represent exons, and gray lines correspond to intronic regions. (**B**) Conserved functional domain composition of GhBXL proteins.

To further investigate the sequence-level conservation and diversity within the BXL gene family, we systematically analyzed the exon–intron structures of cotton *GhBXL* genes (Figure 3A) and the functional domain composition of their encoded proteins (Figure 3B). The results revealed that genes within the same phylogenetic clade often exhibit highly similar structural patterns, supporting their common origin and functional conservation. For instance, the majority of members in Clades A, B, C, E, and F contain 5 to 7 exons with relatively consistent length distributions (Figure 3A). At the protein domain level (Figure 3B), all GhBXL proteins contain three core functional modules: an N-terminal glycoside hydrolase family 3 domain, a C-terminal glycoside hydrolase family 3 domain, and a fibronectin type-III-like domain. Together, these constitute a typical beta-xylosidase catalytic unit. Notably, while the overall domain arrangement is highly conserved across different clades, subtle variations were observed in specific genes. In summary, the *BXL* genes in upland cotton were relatively conserved in terms of their structure and domain distribution.

### 3.4. Chromosomal Distribution and Synteny Analysis of BXL Genes

**Figure 4:**
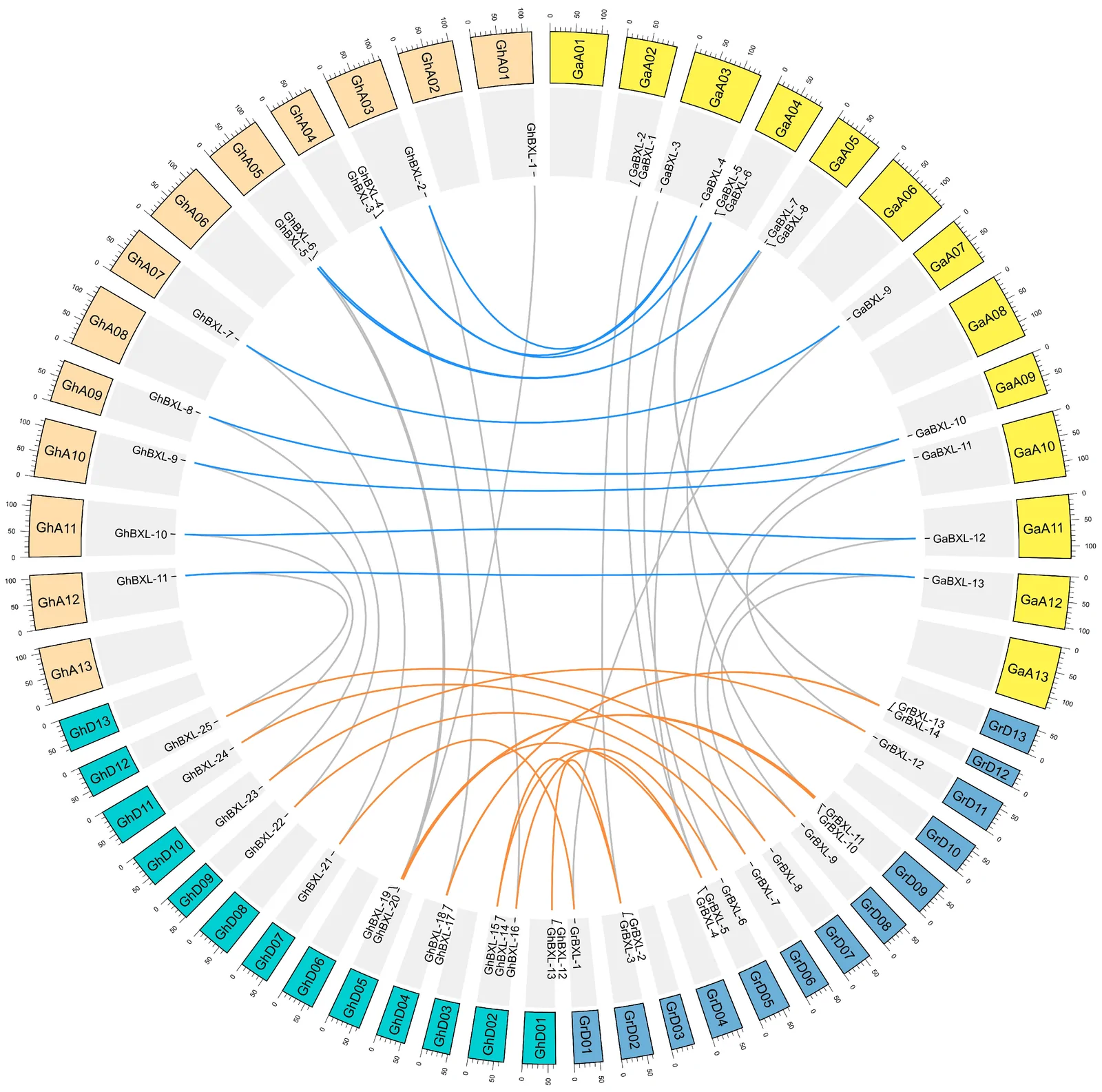
Chromosomal distribution and syntenic relationships of *BXL* genes. Blue lines indicate syntenic relationships between *GaBXLs* and *GhBXLs* (At subgenome), orange lines represent syntenic relationships between *GrBXLs* and *GhBXLs* (Dt subgenome).

To elucidate the evolutionary dynamics of the BXL gene family, we investigated their chromosomal distribution across three cotton species. In *G. arboreum*, the *GaBXL* genes were unevenly distributed across 9 chromosomes. Similarly, in *G. raimondii*, 13 *GrBXL* genes were mapped to 9 chromosomes, showing a dense distribution on chromosome D05. In the allotetraploid *G. hirsutum*, 25 *GhBXL* genes were identified and mapped to 9 chromosomes of the At subgenome and 9 chromosomes of the Dt subgenome. In addition, extensive syntenic blocks containing *BXL* genes were identified between the diploid and tetraploid genomes. Specifically, most *GhBXL* genes in the At subgenome showed clear one-to-one syntenic relationships with *GaBXL* genes, and similarly, the Dt subgenome genes corresponded closely with the GrBXL genes (Figure 4). This high degree of synteny indicates that *BXL* genes have been well-preserved during the evolution from diploid to allotetraploid cotton.

### 3.5. Analysis of cis-Elements in the Promoter Regions of GhBXLs

**Figure 5:**
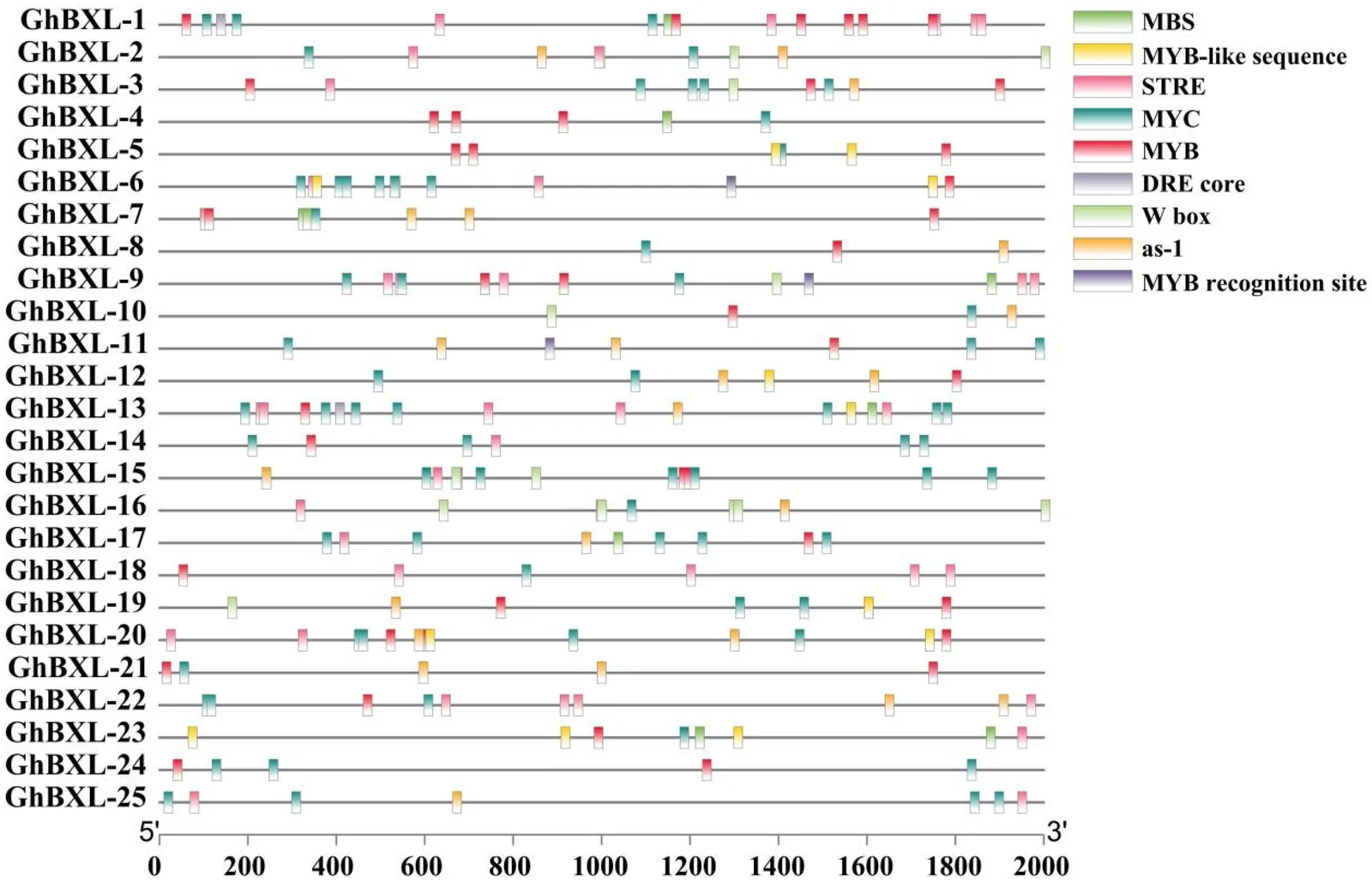
*cis*-acting regulatory element analysis in the promoter regions of the *GhBXL* genes.

To gain insights into the potential regulatory mechanisms and biological functions of *BXL* genes, we analyzed the *cis*-acting elements within the 2000-bp upstream promoter sequences of *GhBXL* genes. The results revealed the presence of multiple types of *cis*-elements. Notably, a variety of stress-related cis-elements were widely identified in the promoter regions of *GhBXL* genes, including STRE, W-box, DRE core, and as-1 (Figure 5). The abundance of these regulatory motifs suggests that GhBXL genes may be involved in abiotic stress-related responses in upland cotton, which requires further experimental validation.

### 3.6. Expression Analysis of GhBXL Genes

**Figure 6:**
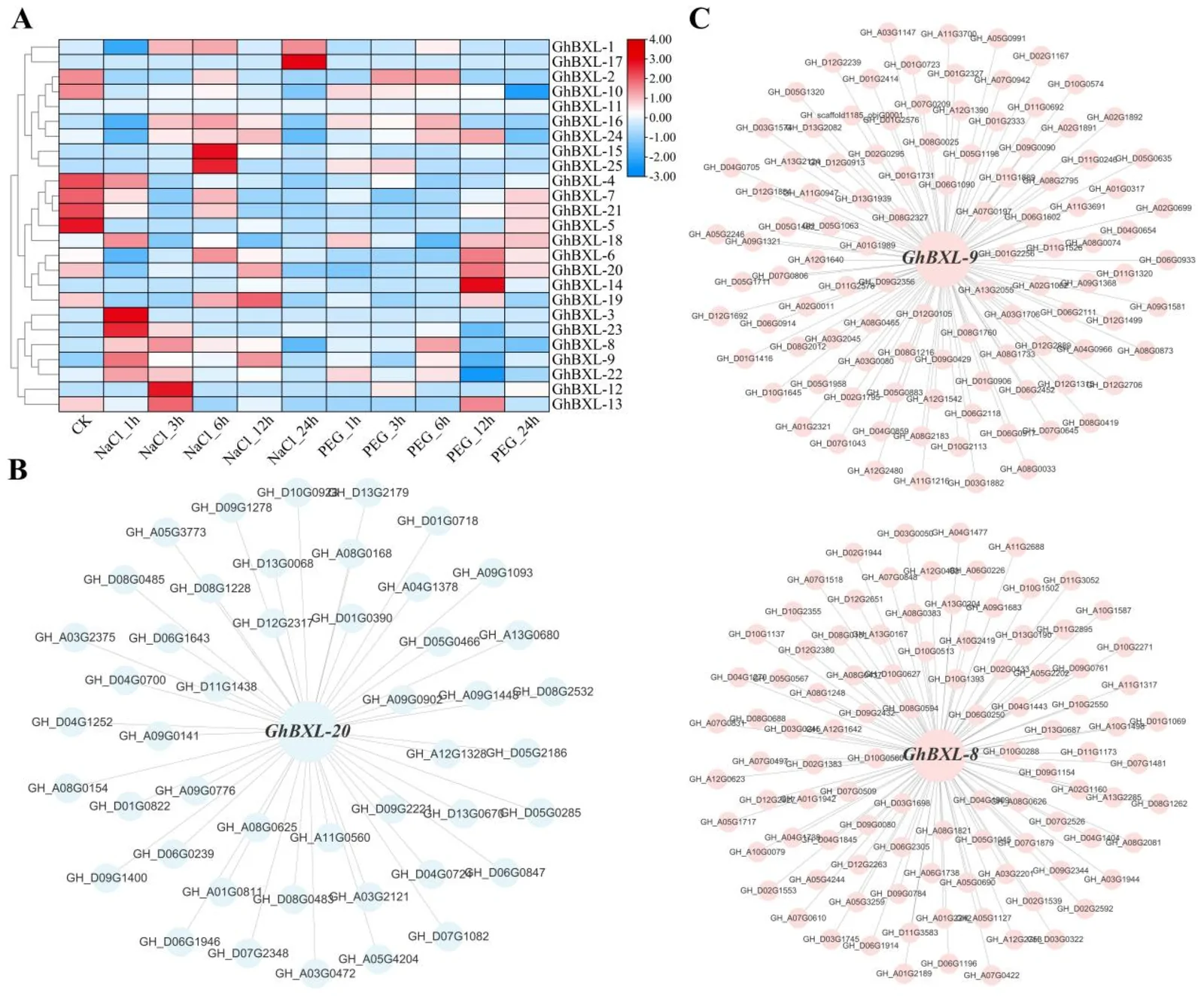
Expression profiles and co-expression network analysis of *GhBXL* genes under abiotic stresses. (**A**) Heatmap showing the expression patterns of 25 *GhBXL* genes under NaCl and PEG treatments at multiple time points (1, 3, 6, 12, and 24 h). The color gradient from blue to red represents relative expression levels from low to high, with CK as the untreated control. The heatmap was generated with TBtools; rows (genes) were hierarchically clustered (Euclidean distance, complete linkage), and expression values were row-normalized. (**B**) Co-expression network centered on *GhBXL*-*20* under salt stress. (**C**) Co-expression networks centered on *GhBXL*-*9* and *GhBXL*-*8* under PEG treatment.

To further investigate the roles of *GhBXLs* in stress resistance, we analyzed their expression profiles using a hierarchical clustering heatmap (Figure 6A). Under PEG stress, the heatmap revealed distinct temporal expression patterns. *GhBXL-18* exhibited strong upregulation specifically at the later stages (12 h and 24 h). In contrast, *GhBXL-8* and *GhBXL-9* showed upregulation primarily at the 6 h time point, indicating an early response. Under salt stress, the heatmap highlighted specific responsive genes. *GhBXL-22* showed significant upregulation at 1 and 3 h, while *GhBXL-20* was upregulated at 12 h (Figure 6A).

Weighted gene co-expression network analysis demonstrated that *GhBXL-8* and *GhBXL-9* were co-expressed with multiple stress-responsive genes under PEG treatment. Transcription factors, including members of the MYB, WRKY, NAC, bZIP, and ERF families, represented the most abundant functional category in the co-expression network. Other prominent functional categories included protein kinases and phosphatases, hormone signaling-associated proteins, and redox-related enzymes, suggesting that these BXL genes may be involved in broader regulatory circuits such as transcriptional regulation and signal transduction under drought stress (Figure 6C, Table S4). It should be noted that co-expression reflects association rather than direct regulatory interaction. Under salt stress conditions, *GhBXL-20* was co-expressed with genes related to cell wall modification and stress adaptation, and transcription factors (including members of the MYB, NAC and bZIP families) were likewise enriched in the *GhBXL-20* co-expression network (Figure 6B, Table S5).

To validate the expression patterns observed in the transcriptome data, we performed qRT-PCR analysis on eight selected *GhBXL* genes under PEG and salt stress. The results confirmed that these genes are responsive to abiotic stress.

**Figure 7:**
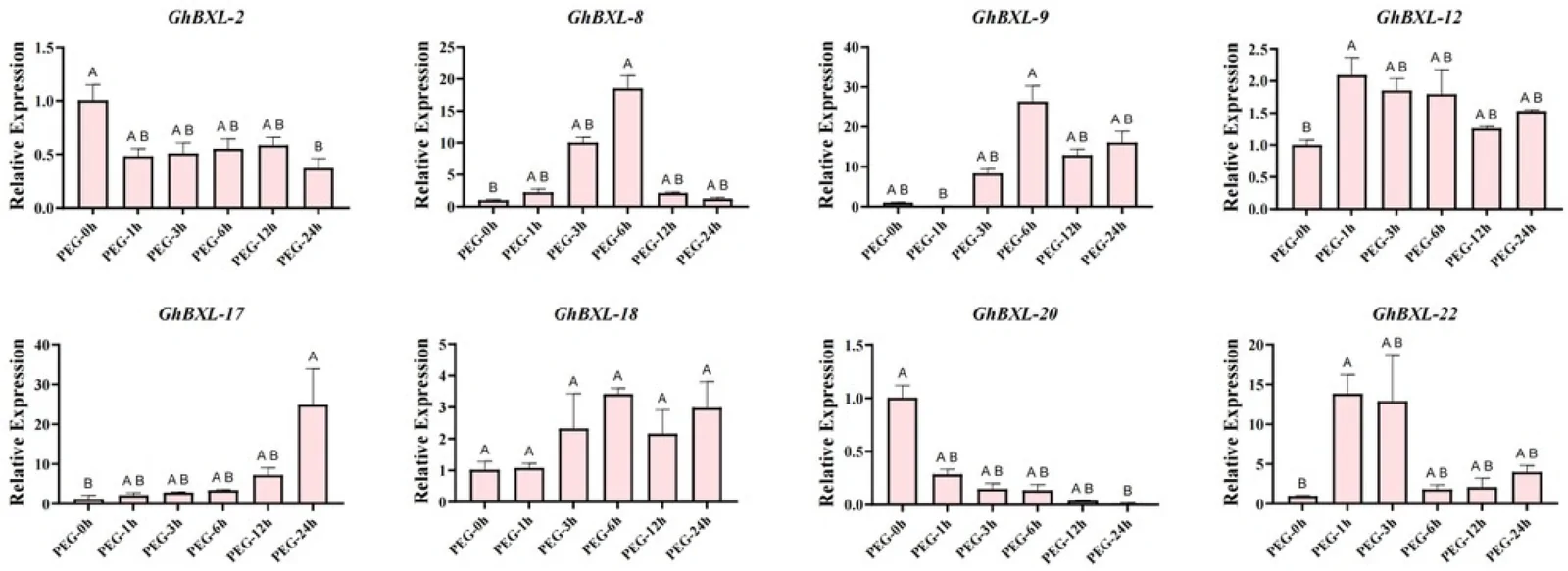
Expression validation of eight *GhBXL* genes under PEG stress by quantitative real-time PCR (RT-qPCR). Each subplot presents the relative transcript abundance of an individual *GhBXL* gene at six time points (0, 1, 3, 6, 12, and 24 h) following PEG treatment. Bars represent the mean ± SD of three biological replicates (*n* = 3). Different uppercase letters above the bars indicate significant differences across time points by the Kruskal–Wallis test followed by Dunn’s multiple comparisons test (α = 0.05).

**Figure 8:**
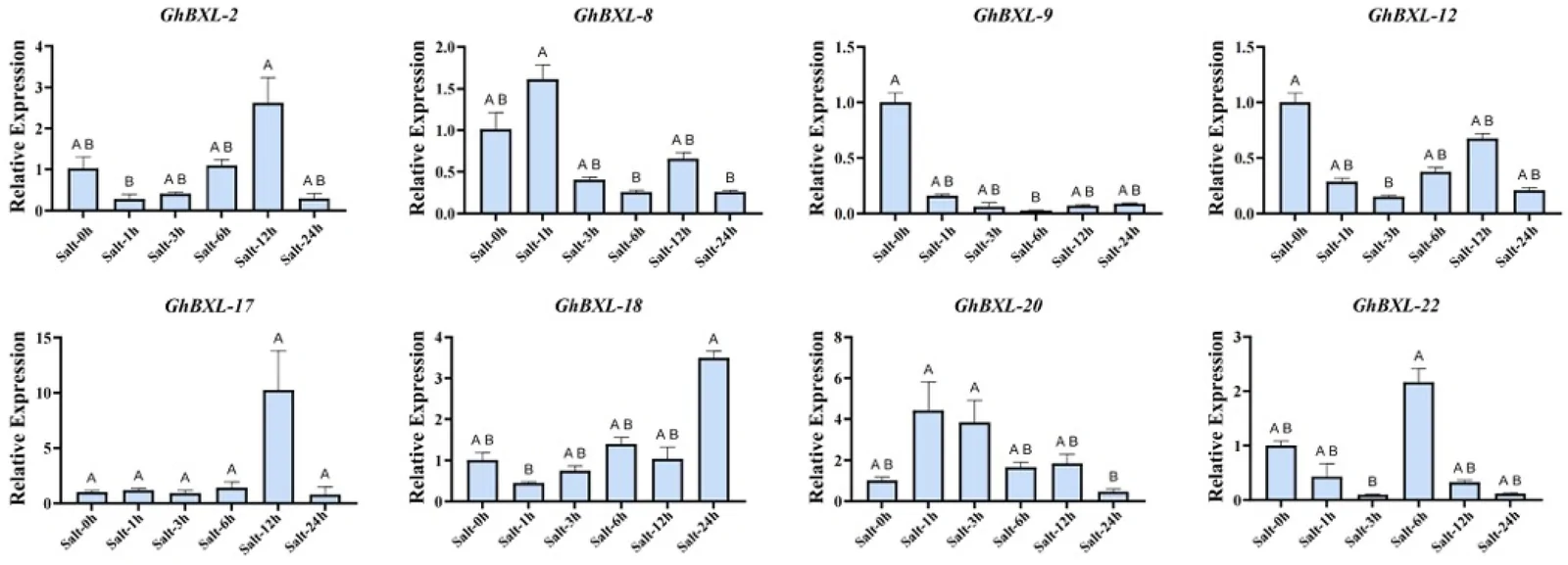
Expression validation of eight *GhBXL* genes under salt stress by quantitative real-time PCR (RT-qPCR). Each subplot presents the relative transcript abundance of an individual *GhBXL* gene at six time points (0, 1, 3, 6, 12, and 24 h) following salt treatment. Bars represent the mean ± SD of three biological replicates (*n* = 3). Different uppercase letters above the bars indicate significant differences across time points by the Kruskal–Wallis test followed by Dunn’s multiple comparisons test (α = 0.05).

Under PEG-simulated drought stress, most of the tested genes showed significant induction. Notably, *GhBXL-17* and *GhBXL-18* exhibited strong upregulation at later time points (12 h and 24 h), with *GhBXL-17* showing a dramatic increase at 24 h. *GhBXL-8* and *GhBXL-9* displayed an early response, peaking at 6 h (Figure 7). Under salt stress, *GhBXL-20* showed a clear induction, although the qPCR trend and RNA-Seq direction differed in timing, both datasets consistently showed the same directional change (Figure 8). The qRT-PCR trends of these genes independently corroborate their stress-responsive expression revealed by the RNA-Seq analysis (Figure 6A), noting that the qRT-PCR treatments were applied at lower intensities (200 mM NaCl; 10% (*w*/*v*) PEG-6000) than the RNA-Seq experiment (400 mM NaCl; 20% (*w*/*v*) PEG-6000).

## 4. Discussion

Cotton, as the world’s most important natural fiber crop, faces continuous threats from abiotic stresses such as drought and salinity, which severely affect its growth, development, and fiber quality [11]. Salt stress inhibits normal cotton growth through multiple pathways including osmotic stress, ion toxicity, and oxidative damage, while drought stress during the reproductive stage leads to boll shedding and significant yield reduction [21]. Therefore, systematic mining of stress-related genes in cotton and elucidation of their underlying mechanisms hold both theoretical and practical value for the molecular breeding of stress-tolerant cotton. Genome-wide gene family identification and analysis can provide comprehensive information on candidate genes from multiple dimensions, including evolutionary history, sequence structure, chromosomal distribution, and expression regulation, thereby laying a foundation for subsequent functional validation and utilization of genetic resources [43,44,45].

In this study, we performed genome-wide identification and systematic analysis of the BXL gene family in allotetraploid cotton (*Gossypium hirsutum*). A total of 25 *GhBXL* genes were identified in the cotton genome, and their phylogenetic relationships, gene structures, conserved domains, chromosomal distribution, promoter cis-acting elements, and expression profiles under drought and salt stress were comprehensively characterized. Cross-species evolutionary analysis across 39 plant species revealed that the BXL gene family is broadly conserved in the plant kingdom, yet gene copy numbers vary substantially among different taxonomic groups. Monocot plants generally possess fewer BXL copies (3–9), whereas dicot plants exhibit a wider range (6–25), a difference that may reflect divergence in genome duplication history and gene retention patterns among lineages. In cotton, the expansion of the BXL gene family may be associated with whole-genome duplication (WGD), consistent with the well-documented paleopolyploidization events in the *Gossypium* genus [46]. The number of BXL genes in allotetraploid *G. hirsutum* and *G. barbadense* is approximately twice that of their diploid progenitor species, a pattern also observed in Brassica napus relative to its diploid ancestors. This additive retention pattern following allopolyploidization suggests that most *BXL* genes were retained after polyploidization events. In cotton, WGD may have been a major contributor of BXL family expansion, whereas in most other species, dispersed or tandem duplications dominate, highlighting the unique evolutionary trajectories of the BXL gene family across different species (Figure 1 and Figure 4). Expression divergence between homeologous A/D BXL pairs was not systematically compared in this study; a detailed homeolog-level analysis would help link the evolutionary and stress-response findings and is an important direction for future work.

Phylogenetic analysis divided the BXL proteins into six clades (A–F). No *G. hirsutum* BXL member was identified in Clade D, which may result from lineage-specific gene loss during cotton evolution or indicate that this subfamily never existed in Malvaceae; broader Malvaceae sampling is required to distinguish these possibilities. In contrast, Clade F is dominated by *G. hirsutum* and also includes a substantial number of monocot genes, suggesting lineage-specific expansions in cotton and grasses. At the structural level, genes within the same clade share similar exon–intron organization, further supporting their common evolutionary origin (Figure 2 and Figure 3). All GhBXL proteins contain three typical domains of GH3 family β-xylosidases: an N-terminal GH3 catalytic domain (PF00933), a C-terminal GH3 domain (PF01915), and a fibronectin type III-like domain (PF14310). This conserved three-domain architecture is consistent with previously reported BXL protein structures in Arabidopsis, soybean, and potato, indicating that these structural units are functionally important for maintaining enzymatic activity [1,9,10].

Promoter *cis*-acting element analysis revealed that the upstream sequences of *GhBXL* genes contain various stress-responsive regulatory motifs, including STRE, W-box, DRE core, and as-1 elements (Figure 5). The presence of these regulatory motifs is consistent with the possibility that GhBXL expression may respond to abiotic stress signals, a notion further supported by transcriptomic and qRT-PCR analyses (Figure 6, Figure 7 and Figure 8). Under PEG-simulated drought stress, different *GhBXL* genes exhibited distinct temporal expression patterns, which suggests that different GhBXL members may participate in distinct phases of the drought stress response. Under salt stress, *GhBXL-22* displayed clear induction, confirming that this gene responds to both stresses and suggesting it as a candidate node that may integrate multiple abiotic stress signaling.

A comparison of our results with published BXL family studies in other species revealed both evolutionary conservation and species specificity in BXL-mediated stress responses. In soybean, 20 out of 29 *GmBXL* genes were coordinately upregulated in roots and leaves under salt stress, indicating a conserved systemic salt-tolerance regulatory mechanism [9]. In rice, *OsBXL1* and *OsBXL3* have been shown to respond to salt–alkali stress [8]. In potato, *StBXL4* was identified as a negative regulator of alkali stress tolerance, with overexpression increasing seedling sensitivity to alkali stress [10]. The divergent regulatory roles of *BXL* genes across different species reflect functional diversification during plant evolution and suggest that BXL isoforms from different lineages may have been recruited into distinct stress adaptation pathways. In cotton, the differentiated temporal expression patterns, together with the presence of stress-responsive cis-elements, support the involvement of *GhBXL* genes in abiotic stress tolerance; however, their specific regulatory mechanisms remain to be elucidated through functional studies. For experimental validation, we prioritized the WGCNA hub genes *GhBXL-8*, *-9*, and *-20*, and additionally included *GhBXL-17*, -*18*, and -*22*, whose transcriptional responses to PEG and salt treatments were particularly strong or distinctive. In future work, we will conduct direct functional validation using virus-induced gene silencing (VIGS), gene overexpression, and CRISPR/Cas9-mediated gene knockout approaches to clarify the roles of specific *GhBXL* members in stress tolerance. In summary, this study systematically reveals the evolutionary characteristics and stress response patterns of the cotton BXL gene family, laying an important foundation for a deeper understanding of the potential roles of cotton BXL genes in stress responses.

## 5. Conclusions

In this study, we performed a genome-wide identification of the BXL gene family in *Gossypium hirsutum*, identifying 25 *GhBXL* genes classified into six phylogenetic clades. Our analyses revealed that the expansion of this family may be associated with whole-genome duplication and allopolyploidization. All GhBXL proteins possess the conserved tripartite GH3 domain architecture, and their promoter regions are enriched in stress-responsive *cis*-elements. Expression profiling under PEG-simulated drought and salt stress, independently confirmed by qRT-PCR, demonstrated distinct temporal response patterns among GhBXL members. Weighted gene co-expression network analysis further identified *GhBXL-8*, *GhBXL-9*, and *GhBXL-20* as hub genes in stress-responsive modules, with their co-expressed partners enriched in transcription factors, kinases, and stress-related proteins. These findings provide a systematic foundation for understanding the evolutionary dynamics and functional roles of *BXL* genes in cotton, and highlight promising candidate genes for future functional validation in stress-tolerance research in cotton breeding programs.

## Data Availability

The RNA-Seq data analyzed in this study are available in the NCBI Sequence Read Archive (SRA) under BioProject PRJNA490626. The genome and annotation files used for gene family identification are publicly accessible from the sources listed in Table S1. Processed data supporting the findings are included in the Supplementary Materials, and further details are available from the corresponding author upon reasonable request.

## Supplementary Materials

The following supporting information can be downloaded at: https://www.mdpi.com/article/10.3390/biology15161419/s1, Table S1. Summary of genome information for the plant species analyzed in this study. Table S2. Primers designed for gene expression analysis by qRT-PCR. Table S3. Characteristics and physicochemical features of BXL family members. Table S4. List of genes and corresponding functional annotations identified in the co-expression network under PEG stress. Table S5. List of genes and corresponding functional annotations identified in the co-expression network under salt stress. Figure S1. Phylogenetic validation of the *Gossypium hirsutum* BXL family assignment.
