# Supplementary material for: Genome-Wide Identification, Evolutionary Analysis, and Expression Profiling of the β-D-Xylosidase Gene Family in Cotton (Gossypium hirsutum) Under PEG-Simulated Osmotic and Salt Stress
Source: Biology (Basel). 2026 Aug 18;15(16):1419. doi: 10.3390/biology15161419 (PMC13509517; doi:10.3390/biology15161419)
Supplement: Supplementary file 1 [file biology-15-01419-s001.zip › Figure S1. Phylogenetic validation of the Gossypium hirsutum BXL family assignment.pdf]

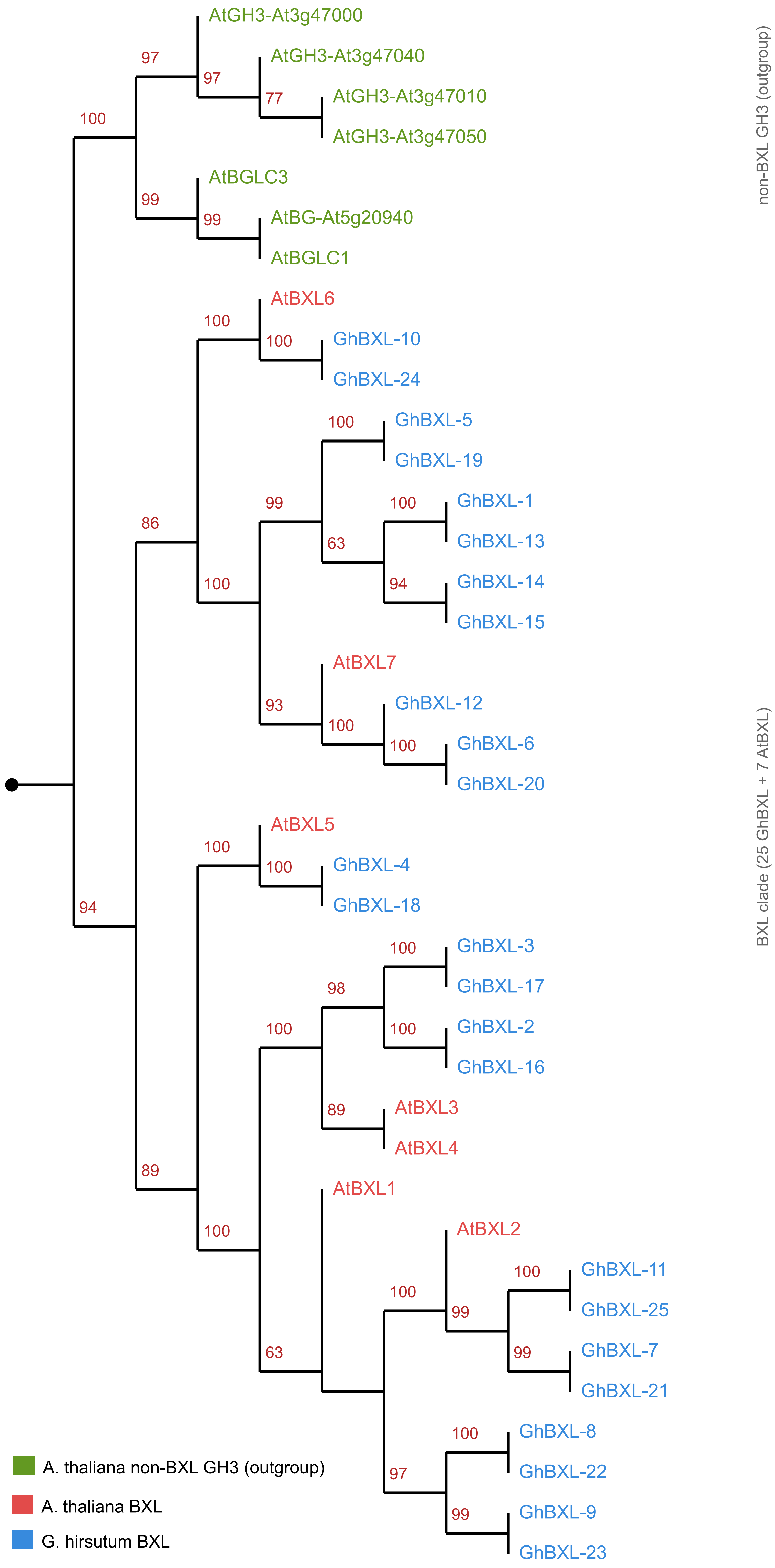
AtGH3-At3g47000
AtGH3-At3g47040
AtGH3-At3g47010
AtGH3-At3g47050
AtBGLC3
AtBG-At5g20940
AtBGLC1
AtBXL6
GhBXL-10
GhBXL-24
GhBXL-5
GhBXL-19
GhBXL-1
GhBXL-13
GhBXL-14
GhBXL-15
AtBXL7
GhBXL-12
GhBXL-6
GhBXL-20
AtBXL5
GhBXL-4
GhBXL-18
GhBXL-3
GhBXL-17
GhBXL-2
GhBXL-16
AtBXL3
AtBXL4
AtBXL1
AtBXL2
GhBXL-11
GhBXL-25
GhBXL-7
GhBXL-21
GhBXL-8
GhBXL-22
GhBXL-9
GhBXL-23
non-BXL GH3 (outgroup)
BXL clade (25 GhBXL + 7 AtBXL)
A. thaliana non-BXL GH3 (outgroup)
A. thaliana BXL
G. hirsutum BXL
